# Acyl-Homoserine Lactone Recognition and Response Hindering the Quorum-Sensing Regulator EsaR

**DOI:** 10.1371/journal.pone.0107687

**Published:** 2014-09-19

**Authors:** Daniel J. Schu, Jessica M. Scruggs, Jared S. Geissinger, Katherine G. Michel, Ann M. Stevens

**Affiliations:** Department of Biological Sciences, Virginia Tech, Blacksburg, Virginia, United States of America; Centre National de la Recherche Scientifique, Aix-Marseille Université, France

## Abstract

During quorum sensing in the plant pathogen *Pantoea stewartii* subsp. *stewartii*, EsaI, an acyl-homoserine lactone (AHL) synthase, and the transcription factor EsaR coordinately control capsular polysaccharide production. The capsule is expressed only at high cell density when AHL levels are high, leading to inactivation of EsaR. In lieu of detailed structural information, the precise mechanism whereby EsaR recognizes AHL and is hindered by it, in a response opposite to that of most other LuxR homologues, remains unresolved. Hence, a random mutagenesis genetic approach was designed to isolate EsaR* variants that are immune to the effects of AHL. Error-prone PCR was used to generate the desired mutants, which were subsequently screened for their ability to repress transcription in the presence of AHL. Following sequencing, site-directed mutagenesis was used to generate all possible mutations of interest as single, rather than multiple amino acid substitutions. Eight individual amino acids playing a critical role in the AHL-insensitive phenotype have been identified. The ability of EsaR* variants to bind AHL and the effect of individual substitutions on the overall conformation of the protein were examined through in vitro assays. Six EsaR* variants had a decreased ability to bind AHL. Fluorescence anisotropy was used to examine the relative DNA binding affinity of the final two EsaR* variants, which retained some AHL binding capability but remained unresponsive to it, perhaps due to an inability of the N-terminal domain to transduce information to the C-terminal domain.

## Introduction

*Pantoea stewartii* subsp. *stewartii* is a phytopathogen that causes Stewart's vascular wilt and leaf blight of maize by producing an abundance of stewartan exo/capsular polysaccharides (EPS) in the xylem of an infected plant [1], [2]. The production of EPS is regulated by the EsaR/EsaI quorum-sensing system. EsaI encodes an acyl-homoserine lactone (AHL) synthase that produces 3-oxo-hexanoyl-L-homoserine lactone constitutively. The transcription factor EsaR functions by binding to target promoters in the absence of AHL, either repressing or activating transcription at low cell densities; derepression or deactivation occurs at high cell densities when AHL associates with EsaR [3]–[5]. Correct temporal expression of the quorum-sensing controlled genes in *P. stewartii* is believed to be important for successful disease progression [4].

EsaR is the best-studied example of a subset of AHL-hindered LuxR homologues, including but not limited to ExpR [6], [7], YenR [8] and EanR [9], which bind to DNA in the absence of AHL and are deactivated when bound to AHL. This mechanism of AHL control is opposite to that of the majority of the LuxR protein family quorum-sensing regulators. To date, only three full-length LuxR homologue structures have been solved [10]–[13] while additional structures of ligand-binding domains are also available [14], [15]. The majority of solved structures are only available in the presence of the cognate AHL or antagonists. Given the lack of structural information available as well as the relatively low sequence similarity of LuxR proteins [16], it is difficult to accurately predict which EsaR residues are responsible for AHL recognition and response.

In comparison to other LuxR proteins, EsaR is relatively stable in the absence of AHL. The protein remains dimeric in both the presence and the absence of AHL and pulse-chase experiments have demonstrated that it does not display differential susceptibility to *in vivo* proteolysis, making EsaR well suited for biochemical analysis [17]. EsaR subfamily members have two unique regions in comparison the majority of LuxR homologues; they have an extended linker region connecting the N-terminal domain (NTD) and the C-terminal domain (CTD) (amino acids 171–178 in EsaR) and they also have an extended CTD (amino acids 237–249 in EsaR) [18] (Figure S1). Some EsaR subfamily proteins repress their own synthesis in the absence of AHL [4], [6], but none of them is known to regulate their cognate synthase genes, which are convergently transcribed from the transcription factor gene [19]. Interestingly all, but one of these proteins preferentially bind to the same AHL, 3-oxo-hexanoyl-L-homoserine lactone; SmaR from *Serratia* is regulated by butanoyl-HSL [19].

This study focused on characterizing the interactions between AHL and EsaR, and thereby interpreting what happens to the protein upon AHL binding. How AHL binding to the N-terminal domain of a LuxR homologue modulates activity of the DNA binding C-terminal domain is an unresolved issue in the quorum-sensing field. Variant forms of EsaR that are more sensitive to AHL have been engineered for use in synthetic biology studies [20]. In the current work, genetic studies were performed to elucidate key amino acid residues involved in the AHL-EsaR interaction through the random generation of EsaR* variants by error-prone PCR. These variants are capable of binding to DNA and repressing transcription of the downstream genes despite the presence of AHL as determined through genetic functional screening, and more quantitatively via β-galactosidase assays. Multiple mutations identified by sequencing were further investigated using site-directed mutagenesis. It was hypothesized that two primary classes of EsaR* variants would be identified from the phenotypic colony screening process; variants that (i) no longer bind AHL or (ii) bind AHL but are no longer responsive and thus still bind to DNA. The DNA binding ability of this second class of variants may be due to resistance to changes in conformation. *In vitro* assays were developed to help distinguish between these two classes of variants.

## Material and Methods

### Strains, plasmids, and growth conditions

Bacterial strains and plasmids used in this study are listed in Table 1. All *lacZ* reporter experiments were performed in *Escherichia coli* Top10 [21] with pRNP-*lacZ*, which encodes the *esaR* promoter fused to *lacZ* [22]. Wild-type and variant forms of *esaR* generated by error-prone PCR and site-directed mutagenesis, are all under control of the P_BAD_ promoter in pBAD22 and their expression was induced with 0.2% L-arabinose. Blue/white screening of the *lacZ* reporter was performed on Luria-Bertani (LB) agar medium with 100 µg/ml ampicillin (Ap100), 50 µg/ml kanamycin (Kan50), 40 µg/ml 5-bromo-4-chloro-3-indolyl-beta-D-galacto-pyranoside (X-gal), and 0.2% L-arabinose. AHL (3-oxo-C6-L-homoserine lactone; Sigma) in 100 µl of acidified ethyl acetate was added to solidified agar via spreading to yield a final concentration of 10 µM, and allowed to evaporate prior to plating of bacteria. Strains for the β-galactosidase assays and western immunoblotting were first grown in LB medium and subsequently subcultured into RM medium (2% casamino acids, 1x M9 salts (10x stock: 128 g/l Na_2_HPO_4_, 30 g/l KH_2_PO_4_, 5 g/l NaCl, 10 g/l NH_4_Cl), 0.4% glucose, and 0.1 M MgCl_2_ containing Ap100 and/or Kan50 when appropriate.

**Table 1:** Strains and plasmids used in this study.

Strain or Plasmid	Relevant Information	References
*E. coli* Top 10	F- *mcrA* Δ(*mrr-hsdRMS-mcrBC*) Ø80d*lacZ*ΔM15 Δ*lac*X74 *deoR recAI araD139* Δ(*ara-leu)7697 galU galK rpsL* (Str^r^) *endA1 nupG*	[21]
*E. coli* MG1655	Wild-type (CGSC no. 7740)	ATCC
**Plasmids**		
pEXT22	IPTG inducible vector, Kan^r^	[32]
pRNP-*lacZ*	pEXT22 with the natural promoter of *esaR* fused to *lacZ*	[22]
pBAD22	Arabinose inducible vector, Ap^r^	[33]
pBAD22-*esaR*	*esaR* ligated into EcoRI site in pBAD22, 15 bp carry over of pGEM vector	[24]
pBADMut1-3 and 5-15^a^	Series of plasmids encoding random mutations in *esaR* producing EsaR* phenotype	This study
pBAD22-#/#^a^ (27/9, 76/26, 94/32, 220/74, 222/74, 241/81, 249/83, 281/94, 302/101, 310G/104, 311/104, 316/106, 398/133, 444/148, 531/178, 607/203, 613/205, 706/236, 728A/243, 728T/243, 734/245)	Series of plasmids encoding site directed mutations producing single amino acid substitutions in EsaR	This study
pDONR201	Gateway entry vector, Kan^r^	Invitrogen
pDEST-HISMBP	Gateway destination vector	[25]
pHMGE	*attb*-His_6_-MBP-TEV-Gly_5_-*esaR*-*attb* (HMGE) in Gateway destination vector	[17]
pHMGE X#X (A32V, A81T, D83E, F94Y, F98Y, S101P, Y104D, I106F)	Series of plasmids encoding HisMBP-Gly_5_-EsaR* variants as indicated	This study
pJW01S	*luxR* divergently transcribed from P*_luxI_* fused to *gfp*	[26]

a for specific substitution see Table 2.

### Generation of EsaR variants through error-prone PCR

Random mutagenesis of *esaR* was performed under conditions similar to those previously published [23]. The level of manganese chloride in the reaction mixture controlled the PCR mutation frequency. Primers were used that directly flank *esaR*, EsaRF-JK anneals to the 5′ end and contains an *Eco*RI site and EsaRR-JK anneals to the 3′ end and contains a *Xba*I site (Table S1). Each PCR reaction contained 1X Thermo Poly Buffer (New England Biolabs (NEB)), 0.2 mM dATP and dGTP, 1.0 mM dCTP and dTTP, 7 mM MgSO_4_, 0.3 mM MnCl_2_, 0.5 µM EsaRF-JK and EsaRR-JK primers, 150 ng/µl of pBAD22-*esaR* template (Table 1), and 1.2 µl *Taq* polymerase (NEB), brought up to 100 µl with dH_2_O. A concentration of 0.3 mM MnCl_2_ was experimentally determined to yield enough PCR product from 16 cycles to produce a visible band within an 0.8% agarose gel. Amplification was performed under the following conditions: 1 cycle: 94°C for 2 min; 15 cycles: 94°C for 30 sec, 50°C for 30 sec, 72°C for 30 sec; 1 cycle: 72°C for two min. After agarose electrophoresis, the desired 800 base pair fragment was purified via a Qiaquick Gel Extraction Kit (Qiagen). A double *Eco*RI and *Xba*I (NEB) digestion was performed on both the mutated *esaR* PCR product and the vector pBAD22 (Table 1), the vector and insert were ligated together using T4 DNA ligase (NEB) and *E. coli* Top10 pRNP-*lacZ* was transformed.

### Screening for strains producing EsaR variants that no longer respond to AHL

*E. coli* Top10 pRNP-*lacZ* strains containing pBAD22 encoding mutagenized *esaR* (Table 1) were phenotypically screened on LB agar medium supplemented with Ap100, Kn50, X-gal and L-arabinose as detailed above. Wild-type EsaR represses transcription of *lacZ* in the absence of AHL, while derepression occurs in its presence; the desired colony phenotype is stably white due to constitutively repressed transcription of *lacZ* +/− AHL. Plasmids from strains with the white phenotype were purified using a Qiaprep Miniprep Kit and sequenced using primers BADVF and BADR (Table S1) at the Virginia Bioinformatics Institute (VBI).

### Quantitative β-galactosidase assays

Strains were grown overnight at 30°C in RM medium with Ap100 and Kan50, but in the absence of arabinose. They were then subcultured to an OD_600_ of 0.05 in the same medium either (a) with 3-oxo-C6-L-HSL and arabinose or (b) with arabinose only, and grown to an OD_600_ of 0.5. Aliquots of cells were stored at -70°C until β-galactosidase assays were performed using a chemiluminescent reporter assay kit (Tropix) and a Beckman Coulter LD 400 microplate reader (Beckman Coulter). β-galactosidase assays for strains expressing the site-directed generated variants were varied slightly by inducing actively growing cultures with arabinose at an OD_600_ of 0.1.

### Western immunoblots

*E. coli* Top10 strains with plasmid constructs of interest were grown overnight in LB and subcultured to an OD_600_ of 0.05 at 30°C. At an OD_600_ of 0.25, arabinose was added to the growing culture to induce protein production and at an OD_600_ of 1.0 one ml aliquots were harvested via centrifugation and diluted in 100 µl 1x standard SDS-PAGE loading buffer [17]. Equal volumes of samples were analyzed via 15% SDS-PAGE and subjected to western immunoblotting with a 1∶500 dilution of polyclonal anti-serum generated against EsaR [24].

### Site-directed mutagenesis

Sequencing revealed that the genes encoding several EsaR* variants had multiple mutations (Table 2). Consequently each individual mutation was separately regenerated via site-directed mutagenesis. Forward and reverse primers containing the desired nucleotide substitutions were designed (Integrated DNA Technologies) and used in PCR reactions in conjunction with upstream vector primer BADVF or downstream vector primer BADR or BADR500 (Table S1). Following the first round of PCR, the desired fragment was gel purified using a Qiaquick Gel Extraction Kit (Qiagen) and a second round of PCR with external primers BADVF and BADR or BADR500 was performed in order to obtain a full length *esaR* gene containing the mutation. Upon completion of the second round of PCR, the full-length esaR* gene and pBAD22 vector were digested with *Nhe*I and *Hind*III (NEB). The resulting fragments were ligated together and transformed into *E. coli* Top10 pRNP-*lacZ*. Blue/white screening and β-galactosidase assays were used to determine EsaR* variants unable to respond to AHL. Plasmids conferring ampicillin resistance were isolated and sequenced (VBI).

**Table 2:** Comprehensive list of mutations achieved in error-prone PCR and the resultant amino acid changes.

Mutant Strain Name	Base Pair Change	Codon Change	Amino Acid Substitution^c^	Polarity/Charge Change for EsaR* phenotype
MUT1	27^b^	CAA to CAT	Q9H	
	94^b^	GCT to ACT	**A32T**	Nonpolar (0) → Polar (0)
	728T^b^	CCG to TTG	P243L	
MUT2	296	TTC to TAC	**F98Y**	Nonpolar (0) → Polar (0)
MUT3	220^b^	TTT to CTT	F74L	
	310G^b^	TAC to GAC	**Y104D**	Polar (0) → Polar (−)
	444^b^	CAG to CAT	Q148H	
MUT4^a^	302^b^	TCC to TAC	**S101Y**	No change
MUT5	95	GCT to GTT	**A32V**	No change
MUT6	301	TCC to CCC	**S101P**	Polar (0) → Nonpolar (0)
MUT7	241^b^	GCC to ACC	**A81T**	Nonpolar(0) → Polar(0)
	316^b^	ATC to TTC	**I106F**	No change
MUT8	310A	TAC to AAC	**Y104N**	No change
MUT9	222^b^	TTT to TTG	F74L	
	296	TTC to TAC	**F98Y**	Nonpolar(0) → Polar(0)
MUT10	311^b^	TAC to TCC	**Y104S**	No change
	398^b^	CAG to CTG	Q133L	
MUT11	281^b^	TTC to TAC	**F94Y**	Nonpolar (0) → Polar (0)
	296	TTC to TAC	**F98Y**	Nonpolar (0) → Polar (0)
MUT12	241	GCC to ACC	**A81T**	Nonpolar (0) → Polar (0)
	316	ATC to TTC	**I106F**	No change
MUT13	76^b^	CTG to ATG	L26M	
	249^b^	GAT to GAG	**D83E**	No change
	281	TTC to TAC	**F94Y**	Nonpolar (0) → Polar (0)
	296	TTC to TAC	**F98Y**	Nonpolar (0) → Polar (0)
MUT14	296	TTC to TAC	**F98Y**	Nonpolar (0) → Polar (0)
	301	TCC to CCC	**S101P**	Polar (0) → Nonpolar (0)
	531^b^	AAA to CAA	K178Q	
	706^b^	GTA to ATA	V236I	
	734^b^	GCG to GTG	A245V	
MUT15	607^b^	GCT to ACT	A203T	
	613^b^	ACG to GCG	T205A	
	728A^b^	CCG to CAG	P243Q	

a Mut 4 was unable to be fully sequenced for unknown reasons; one mutation observed from partial sequence was re-generated through site-directed mutagenesis.

b amino acid substitutions regenerated in isolation via site-directed mutagenesis.

c bold indicates substitutions yielding EsaR* phenotype.

### Construction and purification of histidine (His)_6_-maltose-binding protein-Gly_5_-EsaR* (HMGE*) proteins

HMGE* fusion proteins were constructed through two successive rounds of PCR using an approach similar to that previously described for wild-type protein [17]. The forward primer TEVESAR2 and reverse primer ATTBR (Table S1) were used in the first round to amplify the *esaR* gene from pBAD-EsaR* constructs. The forward primer ATTBTEV and the reverse primer from the first round PCR were then used in the second round of PCR. An 850 bp DNA fragment was recovered containing *attB*-TEV-Gly_5_-esaR*-*attB*. This DNA fragment was then used in the BP and LR reactions of the Gateway cloning system (Invitrogen) using the entry vector pDONR201 (Invitrogen) and the final destination vector pDEST-HISMBP [25] to yield a construct with the P*_lac_* promoter controlling the expression of a His_6_-MBP-TEV-Gly_5_-EsaR* protein (pHMGE*) (Table 1). All constructs were sequenced (VBI).

The HMGE* proteins were purified for *in vitro* studies in a manner similar to that previously described for the wild-type protein [17]. However, the wash buffer contained 300 mM NaCl for the AHL binding and proteolysis assays. For the fluorescence anisotropy experiments, after Ni-NTA column purification, elution fractions were passed over a heparin column (GE Healthcare HiTrap HP, 5 ml). Protein was eluted with a 25 ml linear gradient of 10 mM NaH_2*_PO_4*_H_2_O, pH 7.4 containing 400 mM to 800 mM NaCl. Elution fractions containing HMGE or HMGE* variants were pooled, concentrated and passed through a gel filtration column (GE Healthcare HiPrep 26/60 Sephacryl S-200 HR) equilibrated with HMGE wash buffer (500 mM NaCl, 20 mM HEPES, 10% glycerol, pH 7.4). Fractions containing HMGE or HMGE* were pooled and concentrated with an Amicon ultrafiltration unit with a 10,000 molecular weight cut off filter prior to storage at −70°C.

### *In vitro* AI binding assays

HMGE or HMGE* variants at a final concentration of 2 µM in one mL of resuspension buffer (20 mM HEPES, 300 mM NaCL and 10% glycerol (pH 7.4)) in the presence of 2 µM AHL were exposed to 100 µL of Ni-NTA resin (Qiagen) with gentle rocking for 30 min at 4°C. The protein-bound resin was loaded into a column, which was subsequently washed with 1 mL of resuspension buffer containing 20 mM imidazole. The protein was eluted with a single step gradient of buffer containing 500 mM imidazole. One mL of elution buffer was also exposed to resin by itself and eluted to be used as a blank in quantifying protein concentrations in later steps. The concentration was calculated using OD_280_ readings along with a predicted extinction coefficient for each protein. The protein samples were diluted to a concentration of 92 nM in 1 mL of resuspension buffer. AHL was extracted from the protein by exposing the sample to one mL of acidified ethyl acetate two times, removing the top layer in each case. Three 500 µL aliquots of the extracted AHL in acidified ethyl acetate, for each protein, were placed in separate test tubes, and the acidified ethyl acetate was evaporated from the AHL.

*E. coli* strain MG1655 (wild type (CGSC no. 7740)) pJW0lS (*luxR* divergently transcribed from P*_luxI_* fused to *gfp*) [26] was grown overnight at 30°C in RM medium with 0.4% succinate and Kan50. The strain was then subcultured to an OD_600_ of 0.05 in 200 mL of the same medium, and grown at 30°C to an OD_600_ of 0.25. Five ml was transferred to test tubes containing AHL extracted from HMGE and HMGE* variants and test tubes containing no AHL as a control. The cultures were grown to an OD_600_ of 0.5, then 200 µL of culture was placed in a 96-well optical bottom microtiter plate for the analysis of both fluorescence output (excitation and emission wavelengths of 485 and 535 nm, respectively) and cell density (OD_590_) on a Tecan SpectraFluor Plus plate-reader. The output values were normalized by dividing by the OD_590_ for each sample. The assays were performed as two independent triplicate sets.

### Partial *in vitro* proteolysis

Limited proteolysis of HMGE and HMGE* proteins by thermolysin was performed as previously described [17].

### Fluorescence anisotropy assays

Fluorescence anisotropy assays were developed following established protocols [27], [28]. 5′ TAMRA NHS ester-labeled DNA (PesaR28 TAMRA) and its unlabeled complement (PesaR28R) (Table S1; IDT) were resuspended in annealing buffer (100 mM potassium acetate, 30 mM HEPES [pH 7.5]). The concentration of resuspended oligonucleotides was determined by measuring OD_260_ and using the primer-specific extinction coefficient. For construction of the double-stranded TAMRA-labeled *esa* box (T-esabox), equimolar concentrations of complementary oligonucleotides were annealed by heating to 94°C and slowly cooling to room temperature. Excitation and emission wavelengths of 540 and 590 nm, respectively, were used for experiments. An unlabeled dsDNA *esa* box (esabox) was created as described above using oligonucleotides PesaR28 and PesaR28R (Table S1).

Reagents and materials used for fluorescence anisotropy assays were kept on ice until fluorescence readings were taken. HMGE or HMGE* variants at concentrations ranging from 0 to 100 nM were incubated with 3 nM T-esabox (concentration of dsDNA). The reaction volume and buffer constituents were kept constant (final conditions of 150 mM NaCl, 6 mM HEPES, 17.5 mM Tris, 3% glycerol, pH 7.6) by using a constant amount of protein and HMGE working buffer as well as a constant amount of 25 mM Tris (pH 7.4) and T-esabox. For a given protein concentration, a 90 µl reaction was created from which 40 µl aliquots were placed into two adjacent wells of a Corning 384 well black bottom plate. After 10 and 20 min of temperature equilibration at 25°C, fluorescence anisotropy was measured with a Tecan Infinite F200 Pro fluorometer maintained at 25°C with an integration time of 100 msec and a G factor of 1; the 20 min time point was used to generated reported Kd values since it gave the most consistent results. This experiment was performed in triplicate. For each experiment, background fluorescence was subtracted from the resulting data to obtain Δ fluorescence anisotropy (ΔFA), then the average ΔFA and standard deviation was calculated. The reported dissociation constant (Kd) is the average of the three averages. A fit curve and Kd were generated using XL FIT and Equation 1:
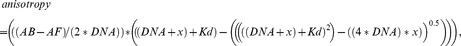
where AB is bound T-esabox, AF is unbound T-esabox, DNA is the concentration of T-esabox and x is the concentration of protein. Similar experiments were performed in the presence of 1 to 8 µM AHL (Sigma), which determined that 1 µM was sufficient for saturation (Figure S2). This concentration of AHL was used for subsequent experiments. As a control, unlabeled *esa* box (0–250 nM) prepared as described above was titrated into solution containing 15 nM HMGE and 3 nM T-esabox maintained in 150 mM NaCl, 6 mM HEPES, 17.5 mM Tris, 3% glycerol, pH 7.6. A fit was generated and a Ki calculated using Equation 2:
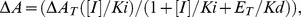
where ΔA is the change in fluorescence anisotropy, ΔA_T_ is the total change in fluorescence anisotropy, [I] is the concentration of unlabeled DNA, Ki is the inhibition constant, E_T_ is the concentration of HMGE, and Kd is the dissociation constant of HMGE [29]. After obtaining ΔFA, the change in anisotropy as the result of binding of the unlabeled DNA (DNAu) was calculated. This was done by subtracting the anisotropy value obtained at [DNAu] = 0 from each data point. The resulting values were used to generate a fit using Equation 2. The same experiment was performed in duplicate.

## Results

### Generating and screening AHL-independent EsaR* variants

To determine which specific amino acids are important for EsaR-AHL interactions that lead to a conformation change in EsaR, *esaR* was randomly mutagenized through error-prone PCR. Following the random mutagenesis, 15 AHL-independent variants (EsaR*) were initially identified that constitutively repressed a *lacZ* fusion, controlled by the *esa* box located at −10 within the *esaR* promoter upstream of *lacZ*, in either the presence or absence of AHL. This repression assay indicates that EsaR* is capable of binding to the *esa* box causing repression of *lacZ* and since this occurs despite the presence of AHL it also serves as a functional screen for EsaR* variants as opposed to other possible types of variants. The constitutive activity of 14 EsaR* variants, for which complete sequence information was obtained, was subsequently verified via β-galactosidase assays (Figure S2A) and a western immunoblot was performed to verify the stability of the variants and determine a relative quantity of EsaR* expressed (Figure S2B). All 14 EsaR* variants were confirmed to be stable and the amount of EsaR* protein produced from the various strains was qualitatively similar.

### Analysis of mutations and amino acid substitutions obtained

Nucleotide sequencing of the genes encoding the 14 EsaR* variants revealed multiple mutations in some isolates. Partial sequence information was obtained for the fifteenth isolate, Mut 4. A total of 25 distinct mutations resulted in 24 individual amino acid substitutions, scattered throughout the protein (Table 2). Since some amino acid substitutions were recovered more than once, the mutagenesis strategy appeared to have approached saturation. Each mutation in *esaR* was regenerated separately via site directed mutagenesis to produce single amino acid substitution variants that were tested individually through the repression assays. Four EsaR* variants were definitively found to have just a single amino acid substitution. Thus 21 amino acid residues were individually replaced via site-directed mutagenesis of *esaR*. Within these 21 mutations, multiple mutations were sometimes found within the same codon, resulting in different amino acid substitutions.

**Figure 1:**
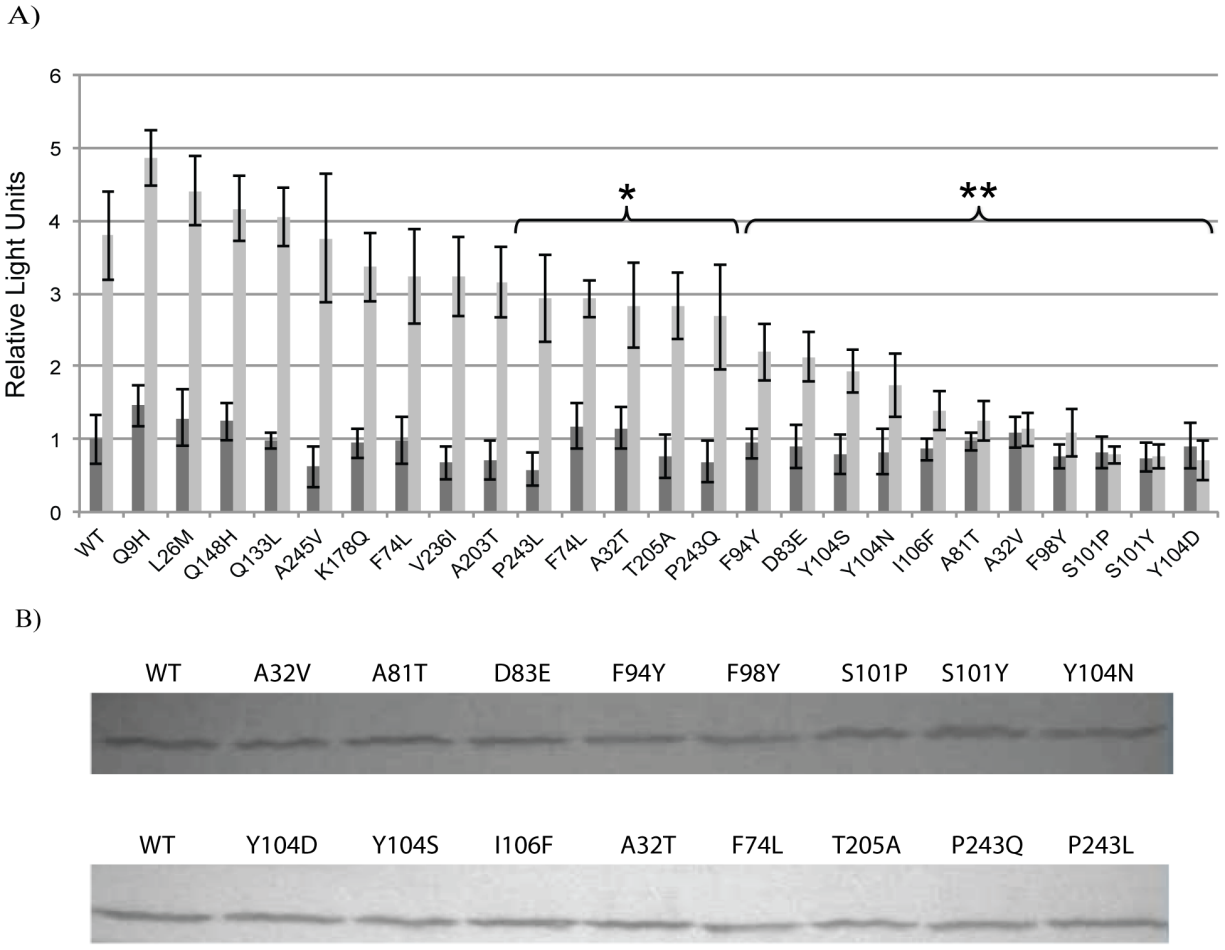
Characterization of EsaR* variants with single amino acid substitutions generated via site-directed mutagenesis. Panel A, Chemiluminescent β-galactosidase assays were performed from two independent experimental samples tested in triplicate with error bars representing the standard deviation of the data, which was normalized to the wild-type control without AHL. Dark grey and light grey bars represent samples without and with AHL, respectively. A statistical comparison of the levels of β-galactosidase produced in the presence of AHL between strains expressing EsaR* variant proteins and the wild-type control is indicated by the brackets highlighting unpaired t test two-tailed P values of <0.004 (*) or <0.0001 (**), respectively. Panel B, western immunoblots demonstrating the stability and relative quantities of 28 kDa wild-type EsaR (WT) and EsaR* variants as indicated. Images are representative of experiments performed in duplicate.

The quantitative β-galactosidase assays of the variants derived through both error-prone PCR and the subsequent site-directed mutagenesis identified 11 mutations encoding amino acid substitutions at residues 32, 81, 83, 94, 98, 101, 104, and 106 that were responsible for an EsaR* phenotype (Figure 1A). Variants at these positions were able to repress transcription in the presence of AHL at a threshold of roughly two-fold (unpaired t test two-tailed P values <0.0001) more than the wild-type control. In addition, these assays also indicated five mutations that produced amino acid substitutions at residues 32, 74, 205 and 243, resulting in an EsaR variant that gave the strain a phenotype intermediate between the wild-type and other EsaR* variants (unpaired t test two-tailed P values <0.004) (Figure 1A). The latter two are located within the C-terminal DNA binding domain of EsaR rather than the N-terminal AHL binding domain. It is interesting to note that four amino acids, 32, 101, 104, and 243 were altered multiple times, resulting in different amino acid substitutions that cause varying degrees of repression. The stability of the EsaR* variants with single substitution at the 11 residues producing the strong to intermediate EsaR* phenotypes was confirmed by western immunoblotting to be relatively equal (Figure 1B).

Eight EsaR* variants (A32V, A81T, D83E, F94Y, F98Y, S101P, Y104D, I106F) were chosen for use in further *in vitro* experiments and N-terminal His-MBP affinity tags were placed on the eight variants to facilitate purification and enhance solubility. Wild-type histidine (His)_6_-maltose-binding protein -Gly_5_-EsaR (HMGE) controls capsule production *in vivo* in an AHL-dependent manner [17]. Assays were developed to differentiate between two potential classes of variants. The first class represents variants with amino acid substitutions that inhibit binding of AHL. The second class represents variants that retain the ability to bind AHL; the residue changes may result in the inability of the protein to undergo normal conformational changes in response to the AHL ligand.

### Ability of EsaR* variants to bind AHL

**Figure 2:**
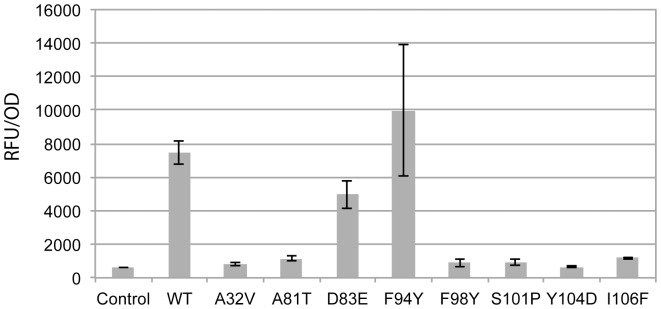
Ability of EsaR* variants to bind AHL *in vitro*. Fluorescence output was measured from an *E. coli* MG1655 reporter strain harboring plasmid pJW0lS, which was exposed to AHL extracted from purified wild-type (WT) or EsaR* protein as indicated. Fluorescence values were standardized by dividing by the final OD_600_ for each sample. The assays were performed as two independent triplicate sets.

An *in vitro* AHL binding assay allowed for direct comparative measure of the ability of the EsaR* variants to bind AHL. In the assay purified HMGE or HMGE* variants were exposed to AHL at a 1∶1 ratio in relation to protein concentration. The proteins were rapidly repurified, and bound AHL was extracted. The extracted AHL was then exposed to recombinant *E. coli* harboring a plasmid that expresses LuxR and contains a *gfp* reporter gene under the control of P*_luxI_*. The *in vitro* AHL binding experiments revealed that two of the eight variants, D83E and F94Y, retained some ability to bind AHL. The six remaining variants appeared to be deficient in AHL binding (Figure 2). Residues 32, 98, 101, 106 identified during the screen, were predicted to be involved in AHL binding based on amino acid alignments with LasR and TraR [13], [14], [16]. When the position of these residues is modeled onto a homology model of EsaR, they cluster around the predicted AHL binding pocket (Figure S3). Therefore, the AHL binding assays suggested that the amino acid substitutions at six of the residues (A32V, A81T, F98Y, S101P, Y104D, I106F) directly or indirectly interfered with AHL binding.

### Conformational analysis of EsaR variants via limited proteolytic digestion

**Figure 3:**
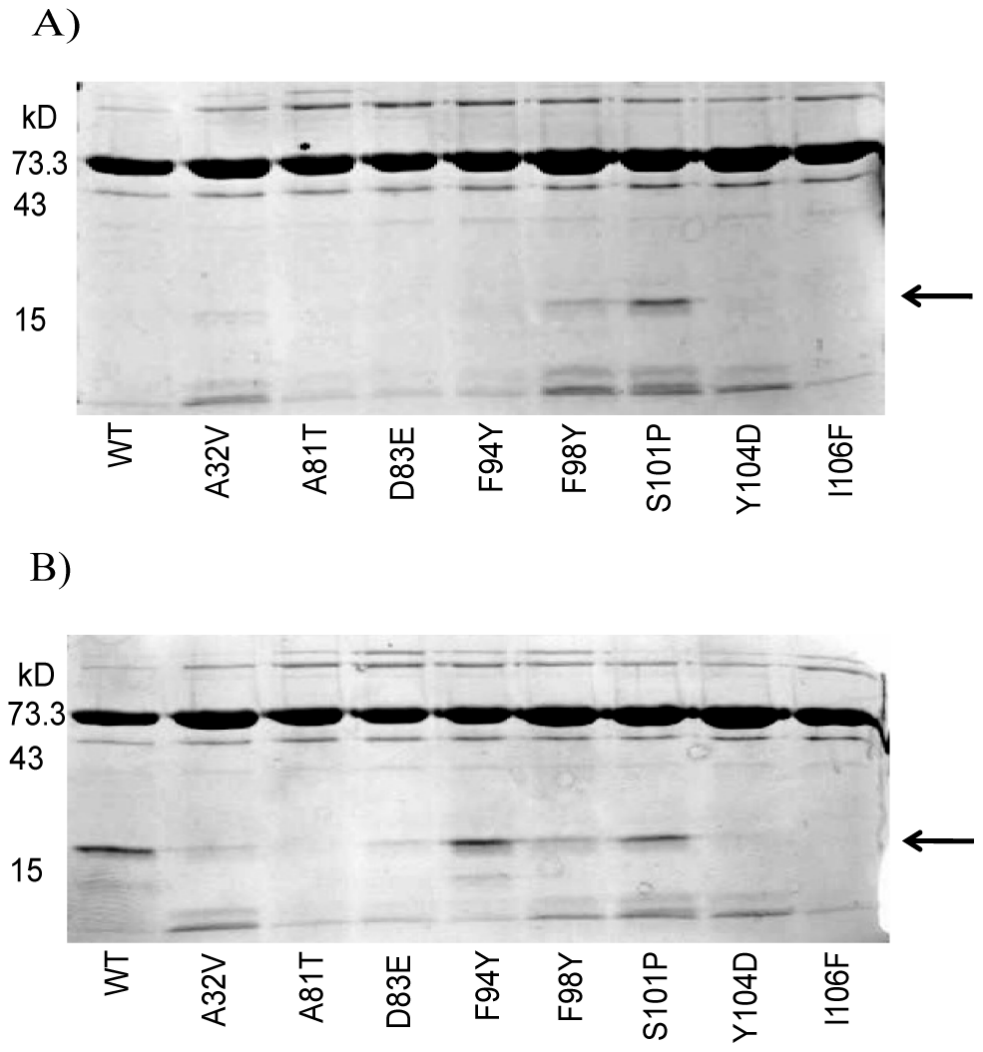
Limited proteolytic digestion of EsaR* variants by thermolysin. A) Patterns of thermolysin cleavage of EsaR* variants in the absence of AHL. B) Patterns of thermolysin cleavage of EsaR* variants in presence of AHL. Arrows indicate bands of interest corresponding to the N-terminal domain of EsaR. The images are representative of experiments performed in duplicate.

An *in vitro* proteolytic assay utilizing the protease thermolysin gives differential digestion pattern of wild-type HMGE in the presence and absence of AHL [17]. Thermolysin preferentially cleaves sites with bulky hydrophobic residues such as isoleucine, valine, alanine, methionine, and phenylalanine. This assay was used to examine structural changes resulting from amino acid substitutions in EsaR. Normally wild-type HMGE has a differential cleavage pattern +/− AHL due to the NTD, which contains the AHL binding pocket, being less susceptible to thermolysin cleavage in the presence of AHL [17]. Of the six EsaR* variants that didn't bind AHL, all six were non-responsive in the proteolysis assays. In both the absence and presence of AHL the three variants A32V, F98Y, and S101P gave a thermolysin cleavage pattern similar to that seen with wild-type EsaR in just the presence of AHL (Figure 3). These proteins are unable to bind AHL and appear to be in a protease-resistant conformation. Conversely, the three variants A81T, Y104D, and I106F gave banding patterns both in the presence and absence of the ligand similar to that produced by wild-type EsaR only in the absence of AHL (Figure 3). These proteins are unable to bind AHL and are in a protease-sensitive conformation.

The final two variants, D83E and F94Y, were unique in that they retained the ability to bind AHL in the *in vitro* binding assays. This phenotype was confirmed with the thermolysin assay, in which the variants demonstrated similar banding patterns compared to wild type +/− AHL suggesting that the variants were capable of interacting with AHL. In the case of the D83E variant, results from both assays suggest that it has a slightly decreased affinity for AHL in comparison to wild type as shown by the levels of Gfp expression in the *in vitro* binding assays (Figure 2), and the intensity of the resistant band with regards to the thermolysin assay (Figure 3). Our data suggest that the D83E and F94Y variants are distinct from the other HMGE* variants by altering the mechanism by which EsaR changes conformations in response to AHL. Interestingly, D83 is quite highly conserved across the EsaR subfamily (Figure S1). Possible explanations for this phenotype are that the mutation in the NTD inhibits signal transduction from the NTD to the CTD precluding release of the protein from the DNA, or that a mutation in the NTD caused a conformational change in the CTD of the protein resulting in tighter DNA binding. To analyze the quantitative DNA binding capability of these two variants in the presence and absence of AHL, fluorescence anisotropy was performed.

### Examination of relative DNA binding affinities of D83E and F94Y EsaR* variants via fluorescence anisotropy

Using fluorescence anisotropy, the Kd of HMGE and three HMGE^*^ variants relative to one another was determined in the presence and absence of AHL. The Kd of HMGE exposed to 1, 2, 4 and 8 µM AHL were first compared, from which it was observed that all were approximately equal (Figure S4). From this it was concluded that of the concentrations of AHL tested, 1 µM AHL was sufficient for saturation and therefore it was used for subsequent experiments. As a control to verify that HMGE was attracted to the *esa* box and not the TAMRA probe, a DNA competition assay was performed. The results demonstrate that anisotropy decreases with increasing concentrations of unlabeled *esa* box, suggesting that the unlabeled *esa* box can compete with the TAMRA-labeled *esa* box (Figure S5).

**Figure 4:**
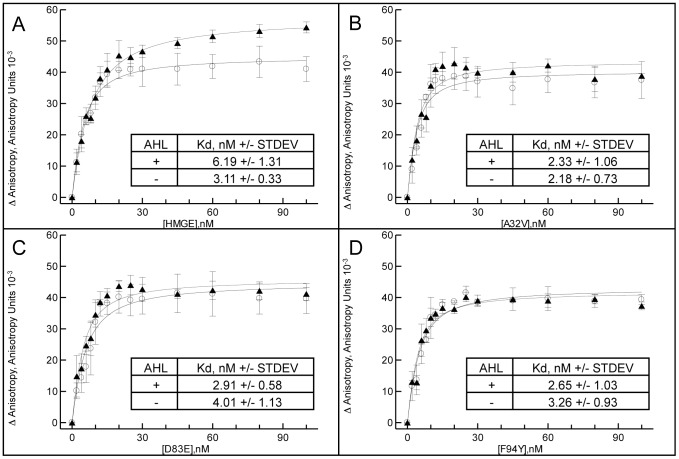
*In vitro* relative binding affinities of HMGE and EsaR^*^ variants. Various protein concentrations (0–100 nM) of A) HMGE, B) A32V, C) D83E, D) F94Y were incubated with 3 nM T-esabox (dsDNA concentration) for 20 min at 25°C in the presence (triangles) and absence (circles) of 1 µM AHL. Fluorescence anisotropy was measured with a Tecan F200 Pro fluorometer with a G factor of 1 and excitation and emission wavelengths of 540 and 590 nm, respectively. Background anisotropy was subtracted and the resulting data were used to generate a fit curve and calculate the apparent Kd. Duplicate samples were analyzed from experiments performed in triplicate. Average of and standard deviation across three experiments for each protein is shown.

The Kd of HMGE in the absence of AHL (3.11+/−0.33 nM) (Figure 4) is in fair agreement with the previously reported Kd of EsaR [30] and is in good agreement with that of other LuxR repressors [6]. In the presence of AHL, roughly a two-fold increase in the Kd was observed (6.19+/−1.31 nM) (Figure 4), indicating that HMGE is responding to the presence of the ligand. Since the P*_luxI_*-gfp assay indicated that A32V is incapable of binding AHL, fluorescence anisotropy was performed on it as a control to verify that the Kd in the presence and absence of AHL remained constant. The constant Kd for A32V in the presence (2.33+/−1.06 nM) versus absence (2.18+/−0.73 nM) of AHL further confirms that DNA binding affinity of A32V is not affected by AHL.

Because two variants (D83E, F94Y) were shown to bind AHL but remain unresponsive to it, it was hypothesized that these two variants were incapable of transducing the NTD ligand detection signal to the CTD. The Kd for D83E in the absence of AHL was 4.01+/−1.13 nM and 2.91+/−0.58 nM in the presence of AHL. Similarly, the calculated Kd for F94Y in the absence of AHL was 3.26+/−0.93 nM and 2.65+/−1.03 nM in the presence of AHL. These values are close to the Kd of the wild-type control without AHL, indicating that theses variants do not bind the DNA with a higher affinity. Because the Kd of these two variants was similar (less than two-fold difference) in the presence and absence of AHL, they appear to have lost the ability to transduce NTD ligand detection to the CTD. The inability of these variants to transduce the signal of AHL detection from the NTD to the CTD, while retaining the ability to bind AHL and DNA suggests that the binding of AHL and binding of DNA is an uncoupled process. Homology modeling to TraR [18] suggested that both these residues are surface exposed on a monomer of EsaR (Figure S3).

## Discussion

The majority of work on the LuxR family of proteins has pertained to AHL-dependent proteins, which require the native AHL for activity and also for purification. EsaR presents a unique opportunity to study the mechanism of AHL-responsiveness used by the LuxR protein family, as it can be purified in the presence and absence of AHL [17]. Here EsaR* variants unresponsive to AHL were isolated. The substitutions resulting in this phenotype may be due to 1) structural changes that cause the AHL binding pocket to refold preventing AHL access to amino acids critical for binding (atoms needed for van der Waals interactions or for hydrogen bonding are no longer present, preventing a stable interaction from occurring), 2) conformational changes that cause the AHL binding pocket to be inaccessible or disappear altogether or 3) the protein locking into a conformation that no longer responds to AHL impeding propagation of the signal from the N-terminal domain to the C-terminal domain preventing release of DNA. Beyond directly impacting AHL binding, 4) C-terminal variants that bind to the DNA with a greater affinity would also be acquired through the screening process.

One set of EsaR* variants (A32V, A81T, F98Y, S101P, Y104D, and I106F) identified in this study was found to impact AHL binding. It appears that AHL associates with a hydrophobic region within EsaR, and by altering residues 32, 98, 101 and 106, which have been shown to be important in either TraR and/or LasR for binding [13], [14], a conformational change is introduced within the N-terminal domain such that the AHL cannot make a stable interaction; critical amino acids for binding are not readily available to AHL. All four amino acids correspond to amino acids of TraR that are engaged in van der Waals interactions or water-mediated hydrogen bonding between AHL and TraR [13] or LasR [14]. Substitutions at residues 81 and 104 created changes in polarity and charge, respectively. This may also have inhibited or repelled AHL binding. Perhaps a bit surprisingly, it appears that both AHL-stimulated and AHL-hindered members of the LuxR family interact with the ligand in a similar manner using conserved amino acid contacts.

In addition to individual amino acids yielding an EsaR* phenotype, it is also possible that certain combinations of amino acid substitutions might produce the same effect. Our random mutagenesis revealed one such variant, Mut15 with A203T, T205A and P243Q substitutions, none of which individually resulted in an EsaR* phenotype, but collectively which did. All three of these substitutions were in the DNA binding CTD and may represent a variant with stronger DNA binding properties than the wild-type protein. Alternatively these mutations could also negate conformational changes, locking the protein into a DNA bound state. Analysis of the additive effect of amino acid substitutions was beyond the scope of this study.

In TraR, AHL appears to be deeply buried within the protein [13]. However in EsaR, the location of this potential binding pocket must be more accessible since AHL modulates the activity of the protein post-translationally. Many of the critical amino acid substitutions identified in this study involved polar amino acid residues. Further *in vivo* studies have shown that as AHL is added exogenously to a growing culture, EsaR responds rapidly by derepressing its own expression [30]. Fluorescence quenching experiments that show EsaR interacts specifically and stoichiometrically with the AHL and surface plasmon resonance revealed that as the concentration of AHL increases, the amount of EsaR capable of forming a complex to the DNA decreases [30].

It has been difficult to biochemically demonstrate the mechanism whereby LuxR homologues transduce a signal from the NTD to the CTD as a result of ligand detection which causes an alteration in DNA binding as a result of AHL detection. It has previously been shown that the CTD of LuxR homologues is not required for AHL binding, suggesting that the functionality of the NTD is independent of the presence of the CTD [31]. In this study, two mutated residues in EsaR (D83E, F94Y) rendered variant forms of EsaR capable of binding AHL but remain unresponsive to it. The results of fluorescence anisotropy assays suggest that these two variants retain the ability to bind DNA in the presence of AHL at levels comparable to the wild type. Therefore, it would appear that these two variants are incapable of transducing the signal of AHL binding from the NTD to the CTD; AHL binding and DNA binding are uncoupled. This is one of the first pieces of biochemical evidence that suggests that a LuxR homologue transduces a signal from the NTD to the CTD as a function of AHL detection. Structural studies of wild-type and variant forms of EsaR in the presence and absence of AHL may give some indication of how this mechanism works.

The detection of and response to AHL by a cognate transcription factor is one of the most fundamental principles of quorum sensing. It is only with a proper understanding of the AHL-transcription factor relationship that quorum sensing-dependent processes will be exploited, holding practical applications. An investigation of the AHL-EsaR relationship may not only hold relevance for *P. stewartii*, but may serve as a model for quorum-sensing systems in an array of other bacteria, especially those employing EsaR homologues.

## Supporting Information

Figure S1 **Amino acid alignment of select members of the LuxR protein family.** The EsaR subfamily is represented by EsaR down through CarR in the list, the other proteins are members of the larger LuxR protein family. Yellow color highlights amino acids responsible for EsaR* phenotype that do not bind AHL, red color highlights amino acids responsible for EsaR* phenotype that retain some ability to bind AHL, * and blue highlights represent single, fully conserved residues,: represents conservation between groups of strongly similar properties, represents conservation between groups of weakly similar properties, green color highlights amino acids associated with the extended linker and C-terminal domain of EsaR, respectively. The alignment was generated using the Universal Protein Resource (www.uniprot.org) with the UniPort Protein Knowledgebase (UniProtKB) align tool's Clustal Omega program.
(TIFF)

Figure S2 Characterization of EsaR* variants generated via random mutagenesis. Panel A, Confirmatory chemiluminescent β-galactosidase assays were performed from one or two independent experimental samples tested in triplicate with error bars representing the standard deviation of the data, which was normalized to the wild-type control. Dark grey and light grey bars represent samples without and with AHL, respectively. Panel B, western immunoblots demonstrating the stability and relative quantities of 28 kDa wild-type EsaR (WT) and EsaR* variants as indicated. Images are representative of experiments performed in duplicate.
(TIFF)

Figure S3 Position of substitutions in EsaR* variants mapped on a homology model of the N-terminal domain of EsaR. Using PyMOL, the side chains of the critical amino acids, 32, 81, 83, 94, 98, 101, 104, and 106, are highlighted on a homology model of EsaR based on TraR [18]: (red) D83E and F94Y (suggested involvement in mechanism of conformational change) (yellow) A32V, A81T, F98Y, S101P, Y104D, and I106F (residues suggested to make direct or indirect interactions with AHL); (green) AHL.
(TIFF)

Figure S4 ***In vitro*** **AHL saturation binding assay.** Various protein concentrations (0–100 nM) of HMGE were incubated with 3 nM T-esabox (dsDNA concentration) for 20 min at 25°C in the presence of 1, 2, 4, 8 µM AHL. Fluorescence anisotropy was measured with a Tecan F200 Pro fluorometer with a G factor of 1 and excitation and emission wavelengths of 540 and 590 nm, respectively. Background anisotropy was subtracted and the resulting data were used to generate a fit curve and calculate the apparent Kd.
(TIFF)

Figure S5 **Unlabeled** ***esa*** **box competition control.** 0–250 nM unlabeled esabox was titrated into reactions containing 15 nM HMGE and 3 nM T-esabox. After incubation at 25°C for twenty min, fluorescence anisotropy was measured with a Tecan Infinite F200 Pro fluorometer with a G factor of 1 and excitation and emission wavelengths of 540 and 590 nm, respectively. Plot shown is the average of experiments performed in duplicate with the Ki obtained from individual experiments. The table indicates the Ki (nM) obtained from each of the two trials and the mean of both trials.
(TIFF)

Table S1 **Primers.**
(DOCX)
