# Supplementary figures and images for: Acyl-Homoserine Lactone Recognition and Response Hindering the Quorum-Sensing Regulator EsaR
Source: PLoS One. 2014 Sep 19;9(9):e107687. doi: 10.1371/journal.pone.0107687 (PMC4169570; doi:10.1371/journal.pone.0107687)

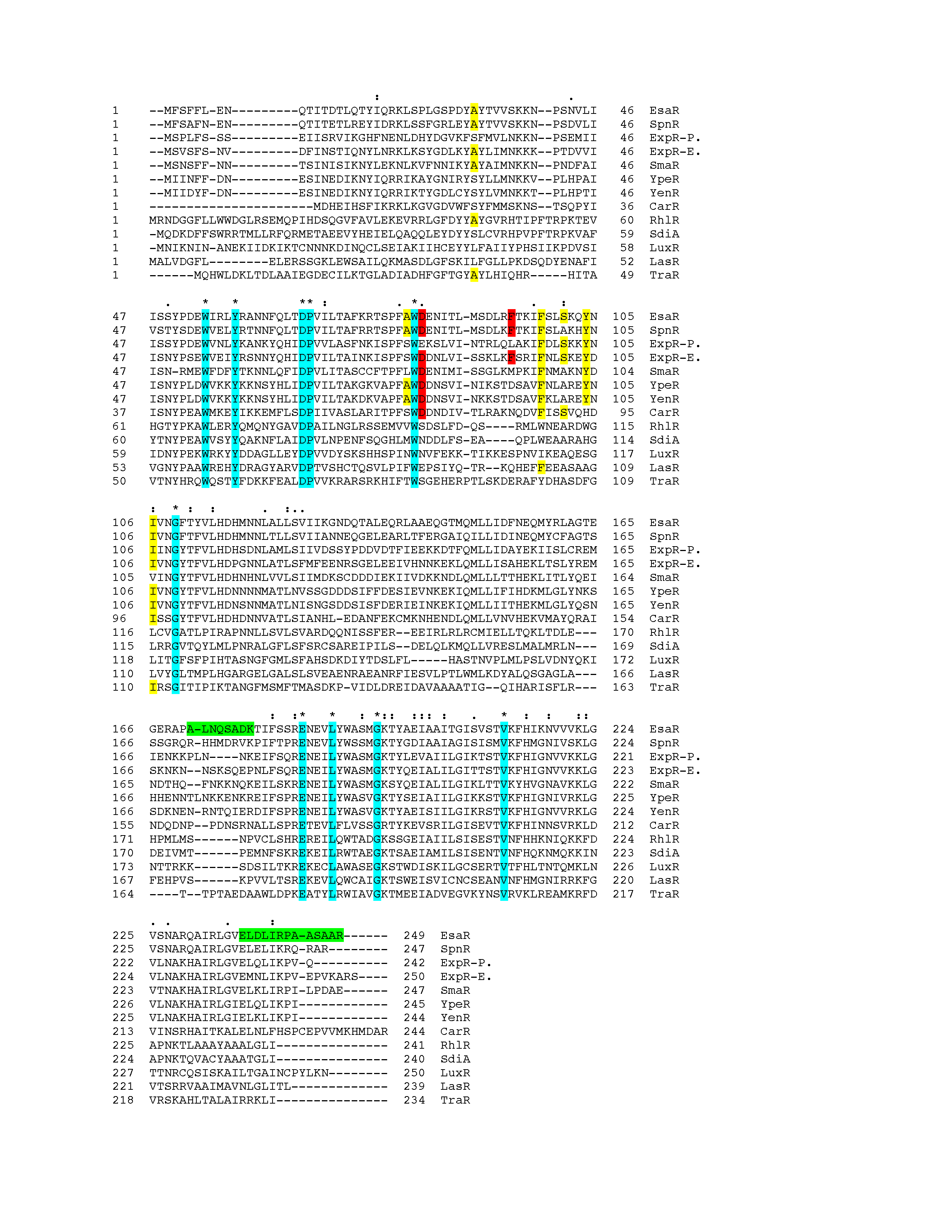

Supplement: Figure S1 — Amino acid alignment of select members of the LuxR protein family. The EsaR subfamily is represented by EsaR down through CarR in the list, the other proteins are members of the larger LuxR protein family. Yellow color highlights amino acids responsible for EsaR* phenotype that do not bind AHL, red color highlights amino acids responsible for EsaR* phenotype that retain some ability to bind AHL, * and blue highlights represent single, fully conserved residues,: represents conservation between groups of strongly similar properties, represents conservation between groups of weakly similar properties, green color highlights amino acids associated with the extended linker and C-terminal domain of EsaR, respectively. The alignment was generated using the Universal Protein Resource (www.uniprot.org) with the UniPort Protein Knowledgebase (UniProtKB) align tool's Clustal Omega program. (TIFF) [file pone.0107687.s001.tiff]

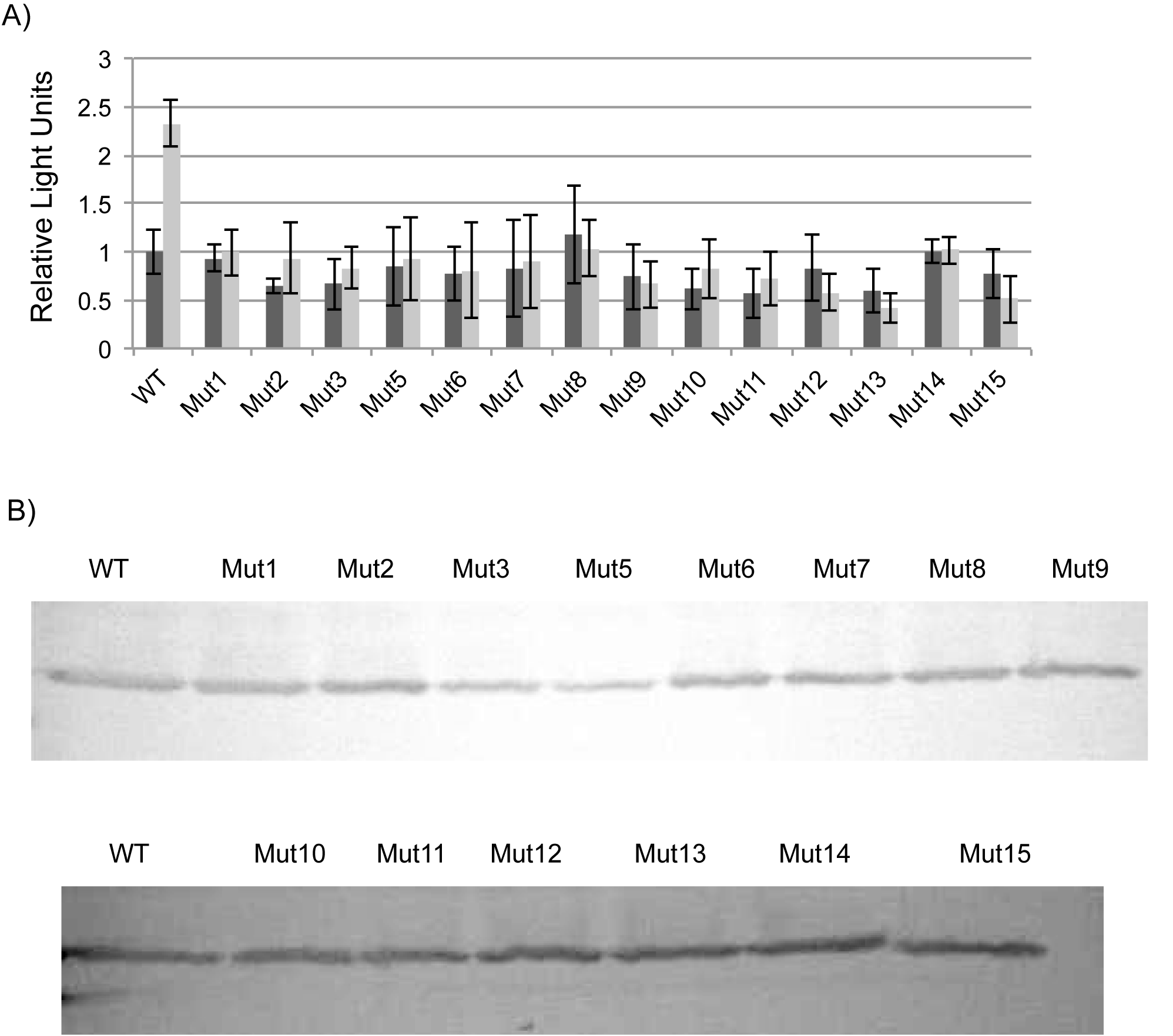

Supplement: Figure S2 — Characterization of EsaR* variants generated via random mutagenesis. Panel A, Confirmatory chemiluminescent β-galactosidase assays were performed from one or two independent experimental samples tested in triplicate with error bars representing the standard deviation of the data, which was normalized to the wild-type control. Dark grey and light grey bars represent samples without and with AHL, respectively. Panel B, western immunoblots demonstrating the stability and relative quantities of 28 kDa wild-type EsaR (WT) and EsaR* variants as indicated. Images are representative of experiments performed in duplicate. (TIFF) [file pone.0107687.s002.tiff]

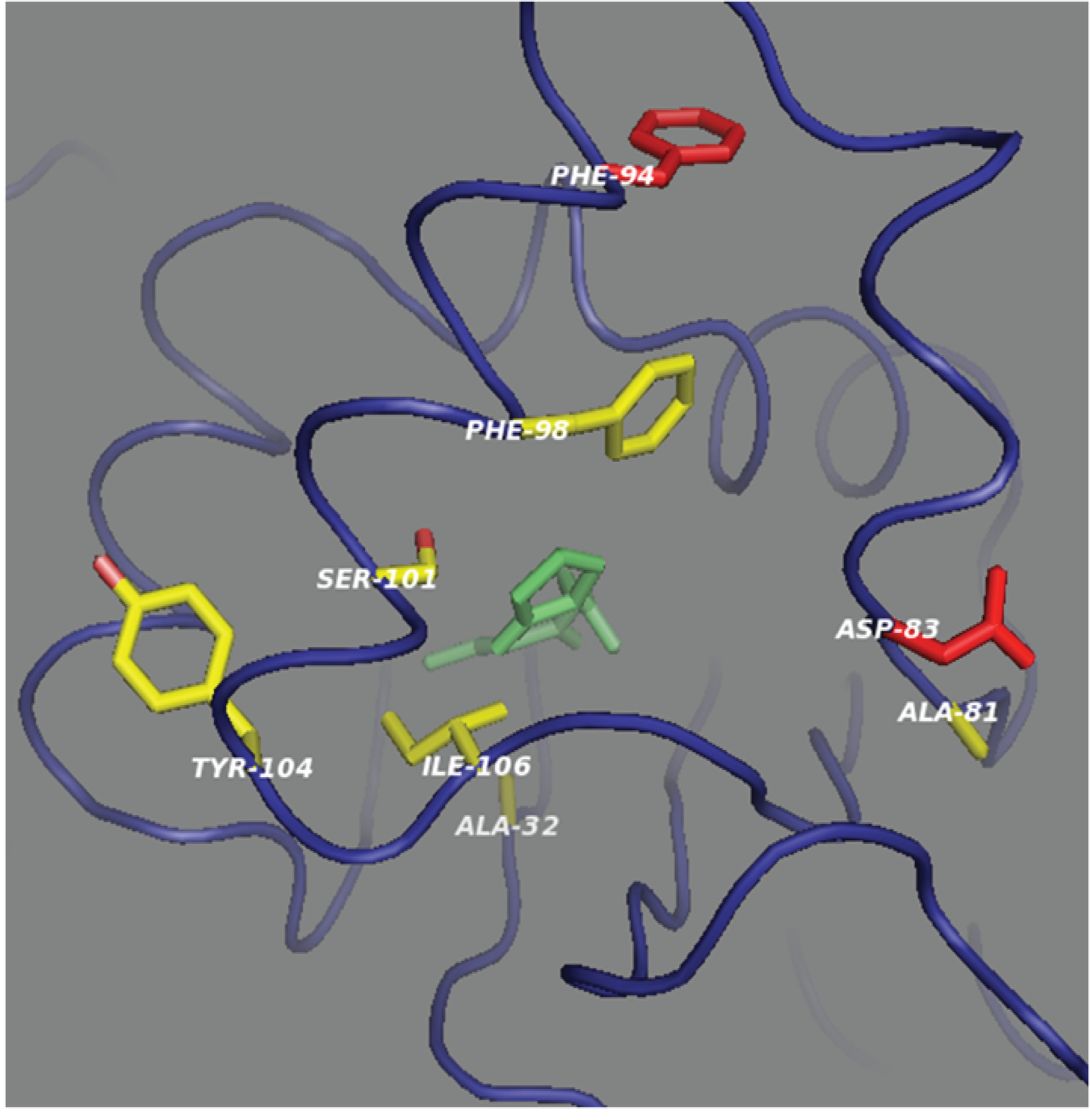

Supplement: Figure S3 — Position of substitutions in EsaR* variants mapped on a homology model of the N-terminal domain of EsaR. Using PyMOL, the side chains of the critical amino acids, 32, 81, 83, 94, 98, 101, 104, and 106, are highlighted on a homology model of EsaR based on TraR [18]: (red) D83E and F94Y (suggested involvement in mechanism of conformational change) (yellow) A32V, A81T, F98Y, S101P, Y104D, and I106F (residues suggested to make direct or indirect interactions with AHL); (green) AHL. (TIFF) [file pone.0107687.s003.tiff]

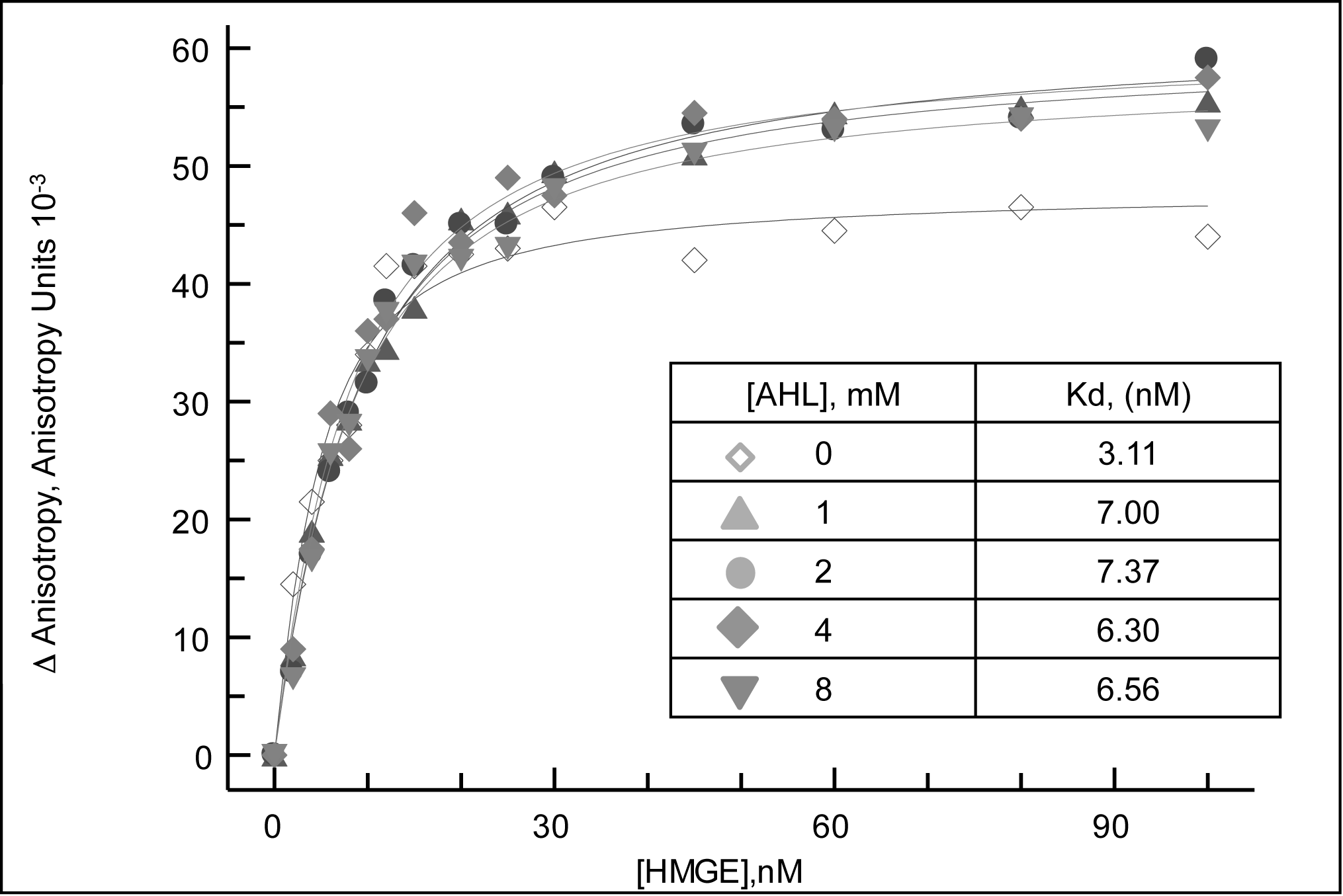

Supplement: Figure S4 — In vitro AHL saturation binding assay. Various protein concentrations (0–100 nM) of HMGE were incubated with 3 nM T-esabox (dsDNA concentration) for 20 min at 25°C in the presence of 1, 2, 4, 8 µM AHL. Fluorescence anisotropy was measured with a Tecan F200 Pro fluorometer with a G factor of 1 and excitation and emission wavelengths of 540 and 590 nm, respectively. Background anisotropy was subtracted and the resulting data were used to generate a fit curve and calculate the apparent Kd. (TIFF) [file pone.0107687.s004.tiff]

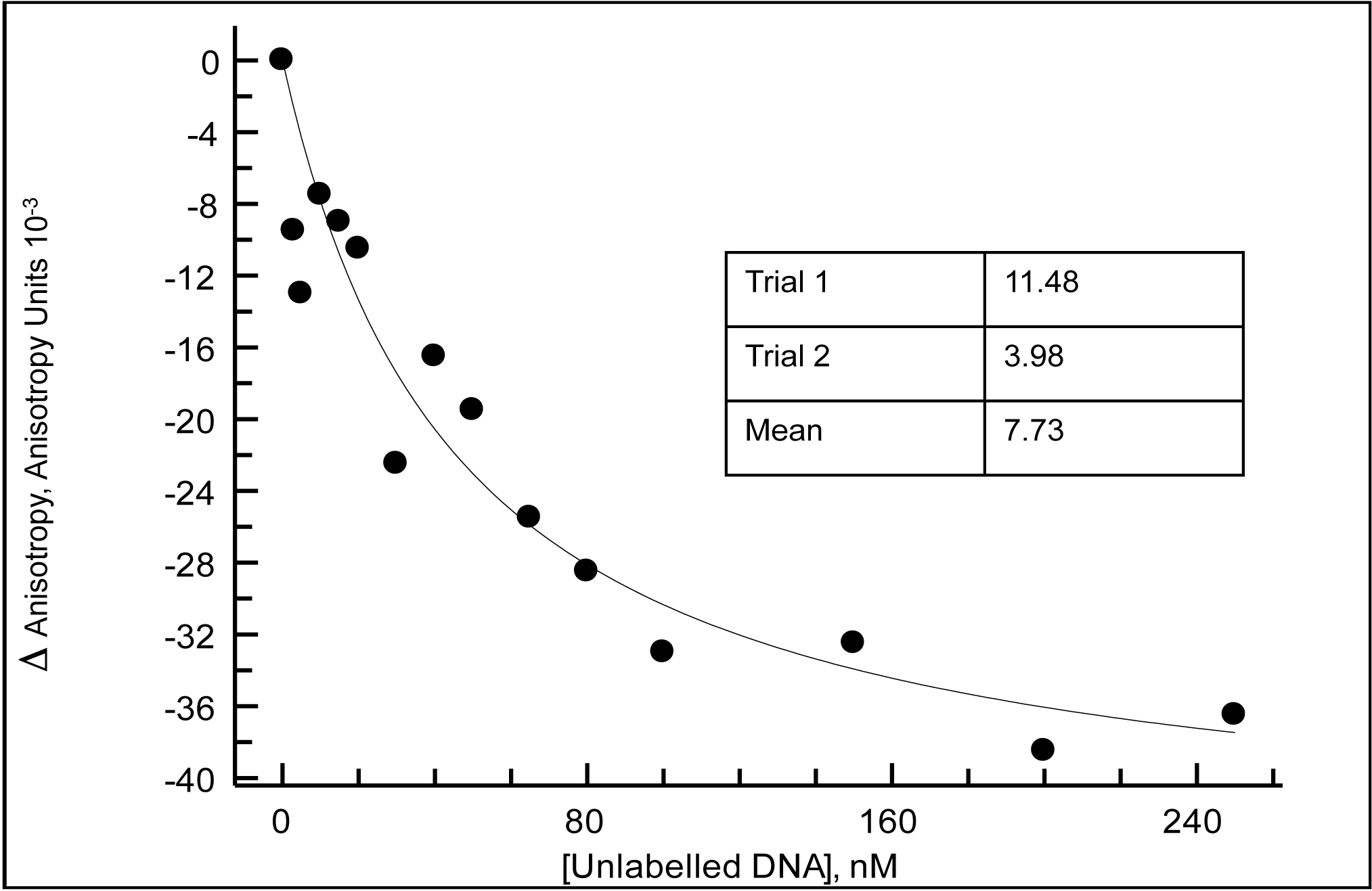

Supplement: Figure S5 — Unlabeled esa box competition control. 0–250 nM unlabeled esabox was titrated into reactions containing 15 nM HMGE and 3 nM T-esabox. After incubation at 25°C for twenty min, fluorescence anisotropy was measured with a Tecan Infinite F200 Pro fluorometer with a G factor of 1 and excitation and emission wavelengths of 540 and 590 nm, respectively. Plot shown is the average of experiments performed in duplicate with the Ki obtained from individual experiments. The table indicates the Ki (nM) obtained from each of the two trials and the mean of both trials. (TIFF) [file pone.0107687.s005.tiff]
