# Supplementary material for: Acyl-Homoserine Lactone Recognition and Response Hindering the Quorum-Sensing Regulator EsaR
Source: PLoS One. 2014 Sep 19;9(9):e107687. doi: 10.1371/journal.pone.0107687 (PMC4169570; doi:10.1371/journal.pone.0107687)
Supplement: Table S1 — Primers. (DOCX) [file pone.0107687.s006.docx]

| **Table S1. Primers** | |
| --- | --- |
| **Primer Name** | **Primer Sequence 5’ to 3’ and References** |
| **Error-prone PCR** | |
| ESARF-JK | CCG**GAATTC**ACCATGTTTTCTTTTTTCC |
| ESARR-JK | GC**TCTAGA**TCACTACCTGGC |
| **Site-directed mutagenesis** |  |
| BADVF | TAACCTTTCATTCCCAGCGGTCG |
| BADR^a^ | CTTCTCTCATCCGCCAAAAC |
| BADR500^b^ | CCCGGCGGATTTGTCCTACTC |
| EsaA27TR | CCTTGAAAATCATACAATAACGGATACGCTTC |
| EsaA27TF | GAAGCGTATCCGTTATTGTATGATTTTCAAGG |
| EsaC76AR | GAAAGTTATCTCCGATGGGTAGTCCGG |
| EsaC76AF | CCGGACTACCCATCGGAGATAACTTTC |
| EsaG94AR | GGGTAGTCCGGATTACACTTACACTGTTG |
| EsaG94AF | CAACAGTGTAAGTGTAATCCGGACTACCC |
| EsaT220CR | CTCACGGCCCTTAAACGCACC |
| EsaT220CF | GGTGCGTTTAAGGGCCGTGAG |
| EsaT222GR | CACGGCCTTGAAACGCACCTC |
| EsaT222GF | GAGGTGCGTTTCAAGGCCGTG |
| EsaG241AR | CTTCGCCGTTTACCTGGGATG |
| EsaG241AF | CATCCCAGGTAAACGGCGAGG |
| EsaT249GR | CGTTTGCCTGGGAGGAGAATATTACGC |
| EsaT249GF | GCGTAATATTCTCCTCCCAGGCAAACG |
| EsaT281AR | GACCTGCGGTACACCAAAATTTTCTC |
| EsaT281AF | GAGAAAATTTTGGTGTACCGCAGGTC |
| EsaC302AR | CACAAAATTTTCTCTTTATACAAGCAATACAACATCG |
| EsaC302AF | CGATGTTGTATTGCTTGTATAAAGAGAAAATTTTGTG |
| EsaT310GR | CTTTATCCAAGCAAGACAACATCGTTAACGGC |
| EsaT310GF | GCCGTTACCGATGTTGTCTTGCTTGGATAAAG |
| EsaA311CR | CCAAGCAATCCAACATCGTTAACGGC |
| EsaA311CF | GCCGTTAACGATGTTGGATTGCTTGG |
| EsaA316TR | CCAAGCAATACAACTTCGTTAACGGCTTTACC |
| EsaA316TF | GGTAAAGCCGTTAACGAAGTTGTATTGCTTGG |
| EsaA398TR | GGCAACGATCTGACTGCGCTGG |
| EsaA398TF | CCAGCGCAGTCAGATCGTTGCC |
| New444R | GGGCACGATGCATATGCTGCTGATTG |
| New444F | CAATCAGCAGCATATGCATCGTGCCC |
| EsaA531CR | CAGAGCGCGGACCAAACG |
| EsaA531CF | CGTTTGGTCCGCGCTCTG |
| EsaG607AR | CCTATGCTGAGATTGCCACTATTACGGG |
| EsaG607AF | CCCGTAATAGTGGCAATCTCAGCATAGG |
| NEW613R | GCTATTGCGGGCATTTCTGTGAGTAC |
| NEW613F | GTACTCACAGAAATGCCCGCAATAGC |
| EsaG706AR | CAGGCTATCAGACTGGGTATAGAACTGGATC |
| EsaG706AF | GATCCAGTTCTATACCCAGTCTGATAGCCTG |
| EsaG728AR | GATCTTATCAGACAGGCAGCGTCAGA |
| EsaG728AF | GCTGACGCTGCCTGTCTGATAAGATC |
| EsaC728TR | CTTATCAGACTGGCAGCGTCAGCG |
| EsaC728TF | CGCTGACGCTGCCAGTCTGATAAG |
| EsaC734TR | CAGACCGGCAGTGTCAGCG |
| EsaC734TF | CGCTGACACTGCCGGTCTG |
| **HMGE variant cloning** |  |
| TEVESAR2 | GAGAACCTGTACTTCCAGGGTGGTGGTGGTGGTATGTTTTCTTTTTTCCTTGAAAATC [17] |
| ATTBR | GGGGACAACTTTGTACAAGAAAGTTGCATTACTACCTGGCCGCTGACGCTC [17] |
| ATTBTEV | GGGGACAACTTTGTACAAAAAAGTTGTGGAGAACCTGTACTTCCAG [17] |
| **Fluorescence Anisotropy** |  |
| PesaR28 TAMRA | TAMRA-TCTTGCCTGTACTATAGTGCAGGTTAAG |
| PesaR28R | CTTAACCTGCACTATAGTACAGGCAAGA [30] |
| PesaR28 | TCTTGCCTGTACTATAGTGCAGGTTAAG [30] |
| a reverse primer for 27, 76, 94, 220, 222, 241, 249, 302, 310, 316, 398, 531 and 607 | |
| b reverse primer for 311, 444, 613, 706, 728 and 734 | |

**REFERENCES**

18. Stevens, AM, Queneau, Y, Soulere, L, von Bodman, S, Doutheau, A. 2011. Mechanisms and synthetic modulators of AHL-dependent gene regulation. Chem. Rev. 111: 4-27.

17. Schu, DJ, Ramachandran, R, Geissinger, JS, Stevens, AM. 2011. Probing the impact of ligand binding on the acyl-homoserine lactone-hindered transcription factor EsaR of *Pantoea* *stewartii* subsp. *stewartii*. J. Bacteriol. 193:6315-6322.

30. Minogue, TD, Wehland-von Trebra, M, Bernhard, F, von Bodman, SB. 2002. The autoregulatory role of EsaR, a quorum-sensing regulator in *Pantoea* *stewartii* ssp. *stewartii*: evidence for a repressor function. Mol. Microbiol. 44:1625-1635.
